# Updates in Thyroid Cancer Surgery

**DOI:** 10.3390/cancers15123102

**Published:** 2023-06-08

**Authors:** Salvatore Sorrenti, Pietro Giorgio Calò

**Affiliations:** 1 Department of Surgery, “Sapienza” University of Rome, 00161 Rome, Italy; 2 Department of Surgical Sciences, University of Cagliari, Monserrato, 09042 Cagliari, Italy; pgcalo@unica.it

This Special Issue of *Cancers* entitled “Updates in thyroid surgery” is a collection of nine articles that covers a wide range of topics, providing a comprehensive picture of the latest developments in thyroid surgery. Esteemed experts in this field have critically analyzed various issues to offer new perspectives, innovative methods, and updated guidelines enhancing the surgical approach and post-operative management of patients with thyroid cancer.

Thyroid cancer, the most common endocrine malignancy, has been exhibiting a steady increase in incidence in the last few years [1]. Thyroid tumors typically arise from follicular cells of the thyroid and commonly present a differentiated phenotype on histology; the most common is papillary thyroid carcinoma, with a favorable prognosis, while less frequent but with an equally favorable prognosis is follicular carcinoma [1]. Poorly differentiated carcinomas and anaplastic carcinomas are rarer, but demonstrate greater aggressiveness and have a significantly worse prognosis [2].

Surgery remains the primary treatment for thyroid cancers; recent years have witnessed the development of new techniques and technologies that enable safer and more effective management [3]. Multidisciplinary approaches and personalized medicine are being employed to improve treatment outcomes for thyroid cancers. By considering each patient’s unique needs and circumstances, these approaches aim to provide more effective and targeted therapies, potentially reducing unnecessary surgeries and improving patient outcomes and quality of life [2].

The advancements in thyroid surgery primarily focus on surgical techniques, aiming to guarantee oncological radicality while achieving excellent aesthetic results and minimizing post-operative complications [4]. Several risk stratification systems, such as the American Thyroid Association (ATA) guidelines, assist in determining the optimal treatment approach [3]. The choice between hemithyroidectomy and total thyroidectomy is a primary consideration. Hemithyroidectomy allows for safe treatment, with fewer complications and the possibility of maintaining adequate thyroid function; at the same time, however, it does not affect the possibility of a second completion surgery when needed [5]. Surgical intervention remains the preferred treatment for lymph node metastases in papillary thyroid carcinoma, with laterocervical dissection being the recommended method for local disease control; thorough preoperative assessment and precise surgical techniques are crucial for minimizing complications, and technology has played an increasingly significant role in this regard, simplifying the preservation of nerves, blood vessels, and parathyroid glands [6]. Minimally invasive surgery techniques, robotic surgery, and ablative therapies such as radiofrequency ablation have joined traditional surgery. Notably, transoral thyroidectomy, known as TOEVA, allows the thyroid gland to be removed through a hidden incision in the oral vestibule with excellent aesthetic results and a surgical outcome comparable to open surgery [7].

In addition to the most suitable approach, the use of surgical devices and technology to support surgical techniques plays a crucial role in ensuring safer surgery. Among these, intraoperative neuromonitoring has been widely employed in thyroid surgery, facilitating the early identification of recurrent laryngeal nerve injuries and improving the management of this complication [8]. It is also possible for the surgeon, in patients affected by papillary carcinoma of the thyroid with lymph node metastases, to be supported by intraoperative diagnostics; indeed, Di Meo et al. demonstrated how intraoperative ultrasound locates the laterocervical lymph nodes, allowing a more selective and reliable neck dissection and ensuring the oncological radicality [9].

Over the years, the ability to diagnose thyroid tumors early has improved more and more, enabling the selection of patients worthy of further investigations and thus increasingly limiting the number of unnecessary procedures. Advanced diagnostic tools can support specialists in patient assessment, risk stratification, and the selection of appropriate therapeutic procedures. Diagnostic imaging techniques have evolved and been refined over the years, from basic ultrasound to contrast-enhanced ultrasound (CEUS), elastosonography, and the application of artificial intelligence and machine learning for thyroid nodule characterization [10].

The advancement of surgical techniques, continuous improvement in diagnosis and alternative or adjuvant therapies must be accompanied by a deep understanding of cancer biology. In particular, the treatment of thyroid cancer is increasingly evolving towards precision and personalized medicine, allowing therapeutic strategies to be tailored to each patient’s needs and the biological and molecular characteristics of the tumor. Extensive research is ongoing to identify specific biomarkers [11], genetic alterations [12,13,14,15] or risk factors [16,17] that can predict disease behavior, underpin cancer development and progression, or aid in prevention and treatment [1]. For example, surgery represents the only option for the treatment of non-responsive radioiodine cancers and avoids or delays the onset of local complications, although in most cases it is not possible to achieve either cure or local clearance; in fact, there is still a lack of early biological markers and systemic treatments that allow for the safe and definitive treatment of these patients [18].

In conclusion, innovations in thyroid surgery have led to significant advancements in the treatment of thyroid cancer. Minimally invasive techniques, the utilization of technologies, and the introduction of biomaterials have enhanced the safety and efficacy of surgical interventions, resulting in reduced complications and improved aesthetic outcomes. Simultaneously, innovations in diagnostic imaging and advanced imaging tools and ongoing discoveries in molecular biology have made it possible to optimize treatments, with a view to personalized therapy.
